# Familial Alzheimer’s disease mutations at position 22 of the amyloid β-peptide sequence differentially affect synaptic loss, tau phosphorylation and neuronal cell death in an *ex vivo* system

**DOI:** 10.1371/journal.pone.0239584

**Published:** 2020-09-23

**Authors:** Christian Tackenberg, Luka Kulic, Roger M. Nitsch

**Affiliations:** 1 Institute for Regenerative Medicine, University of Zurich, Schlieren, Switzerland; 2 Neuroscience Center Zurich, University of Zurich and ETH Zurich, Zurich, Switzerland; Nathan S Kline Institute, UNITED STATES

## Abstract

Familial forms of Alzheimer’s disease (AD) are caused by mutations in the presenilin genes or in the gene encoding for the amyloid precursor protein (APP). Proteolytic cleavage of APP generates the β-amyloid peptide (Aβ), which aggregates into amyloid plaques, one of the major hallmarks of AD. APP mutations within the Aβ sequence, so-called intra-Aβ mutations, cluster around position E693 of APP, which corresponds to position E22 in the Aβ sequence. One of these mutations is the Osaka mutation, E693Δ, which has unique aggregation properties with patients showing unusually low brain amyloid levels on amyloid PET scans. Despite intense research on the pathomechanisms of different intra-Aβ mutants, our knowledge is limited due to controversial findings in various studies. Here, we investigated in an *ex vivo* experimental system the neuro- and synaptotoxic properties of two intra-Aβ mutants with different intrinsic aggregation propensities, the Osaka mutation E22Δ and the Arctic mutation E22G, and compared them to wild-type (wt) Aβ. Experiments in hippocampal slice cultures from transgenic mice were complemented by treating wild-type slices with recombinantly produced Aβ40 or Aβ42 containing the respective intra-Aβ mutations. Our analyses revealed that wt Aβ and E22G Aβ, both recombinant and transgenic, caused a loss of dendritic spines along with an increase in tau phosphorylation and tau-dependent neurodegeneration. In all experiments, the 42-residue variants of wt and E22G Aβ showed stronger effects than the respective Aβ40 isoforms. In contrast, E22Δ Aβ neither reduced dendritic spine density nor resulted in increased tau phosphorylation or neuronal cell death in our *ex vivo* system. Our findings suggest that the previously reported major differences in the aggregation kinetics between E22G and E22Δ Aβ are likely reflected in different disease pathomechanisms.

## Introduction

Alzheimer’s Disease (AD) is the most common age-related neurodegenerative disorder. The major histopathological hallmarks are the presence of extracellular aggregates of Aβ in amyloid plaques and intracellular neurofibrillary tangles containing hyperphosphorylated tau as major component. According to the amyloid hypothesis Aβ aggregates, especially oligomers, are the driver of AD pathology [1]. Aβ is generated by proteolytic processing of APP by the β- and γ-secretase complexes. Familial forms of AD are caused by mutations in presenilin 1 or presenilin 2, the catalytic subunits of the γ-secretase complex, or by mutations in APP itself. APP mutations can have various effect on APP processing, depending on their location. Mutations around the β-secretase cleavage site, such as the APP_swe_ mutation K670N/M671L, increase the general production of Aβ, whereas mutations near the γ-secretase cleavage site shift the processing towards longer isoforms of Aβ, such as Aβ42, which is thought to be more toxic than the shorter Aβ40 variant [2]. Mutations within the Aβ sequence of APP alter the aggregation propensity of Aβ. A special cluster of these intra-Aβ mutations is localized at position E693 of APP, corresponding to position E22 in the Aβ sequence, a hydrophobic core of the peptide. Mutations at this position include the Dutch (E693Q) [3], Italian (E693K) [4], Arctic (E693G) [5] or Osaka (E693Δ) mutation [6].

The Arctic mutation E693G was discovered in 2001 in a Swedish family showing clinical symptoms of early-onset AD. Carriers of this mutation show decreased Aβ42 and Aβ40 levels in plasma [5] and low PiB PET retention while cerebral glucose metabolism and CSF levels of Aβ42, total and phosphorylated tau are highly pathologic [7]. The Osaka mutation E693Δ, discovered in 2008, shows a recessive pattern of inheritance that causes AD-like symptoms despite very low PiB PET signals [6]. As synthetic E22Δ Aβ was shown not to form fibrils, it was hypothesized that this mutation favours oligomerization causing AD in the absence of fibril/plaque formation [6]. However, the literature on the aggregation propensity of the E693Δ mutation is controversial as subsequent studies, including our own research using recombinant Aβ peptides, showed fibril formation by E22Δ Aβ [8–11]. Compared to wt and E22G, recombinant E22Δ Aβ 40 and Aβ 42 had unique aggregation characteristics in thioflavin T aggregation assays with E22Δ Aβ42 forming SDS-resistant amyloid fibrils direct after reconstitution, while fibril growth occurred non-exponentially. In contrast, E22Δ Aβ40 aggregation was characterized by an exponential growth phase [8]. These findings were supported *in vivo*. Extracellular accumulation of insoluble Aβ occurred in E22ΔAβ transgenic mice as leptomeningeal cerebral amyloid angiopathy at the age of 24 months, while intraneuronal Aβ oligomers were detected already at 3 months. In contrast, arcAβ transgenic mice, expressing E22G Aβ and Tg2576 mice, which express wt Aβ, accumulated detergent-insoluble, fibrillar aggregates already at 6 month (arcAβ mice) and 15 months (Tg2576) of age [8].

Besides several studies on the mechanisms of E22Δ Aβ toxicity, a direct comparison to wt and E22G Aβ for its neuro- and synaptotoxic properties has not been performed yet. Therefore, we analyzed the effects of recombinant E22Δ, E22G and wt Aβ40 and Aβ42 on neuronal survival and dendritic spine densities in an *ex vivo* experimental paradigm in hippocampal slice cultures. Treatment of wild-type slices with recombinant Aβ was directly compared to slices obtained from the respective transgenic mouse models.

We found that both recombinant wt and E22G Aβ42 induced loss of dendritic spines, increased tau phosphorylation and caused a tau-dependent cell death in wild-type slices. This was confirmed in slices from Tg2576 and arcAβ transgenic mice. In contrast, neither induction of toxicity nor an increase in tau phosphorylation were observed in slices treated with E22Δ Aβ40 or Aβ42 or in slices from E22ΔAβ transgenic mice, suggesting differential disease pathomechanisms for distinct intra-Aβ mutants.

## Materials and methods

### Animals

ArcAβ mice and E22ΔAβ mice were obtained as previously described [8, 12]. Tg2576 mice (B6;SJL-Tg(APPSWE)2576Kha) were obtained from Taconic Biosciences, Rensselaer, NY, USA. Details on respective APP mutations are displayed in Table 1. All animal experiments were performed in compliance with Swiss national guidelines and were approved by the veterinarian office of the Canton of Zurich. Animals were housed in groups. For preparation of hippocampal slice cultures P6-P8 mice were sacrificed by decapitation.

**Table 1:** APP/Aβ mutants used in this study.

	Tg2576 mice [16]	arcAβ mice [12]	E22ΔAβ mice [8]	recombinant wt Aβ	recombinant E22G Aβ	recombinant E22Δ Aβ
APP/Aβ mutations	APP_swe_ (K670N + M671L)	APP_swe_ (K670N + M671L); APP_arc_ (E693G (= E22G))	APP_swe_ (K670N + M671L); APP_osaka_ (E693Δ (= E22Δ))	no mutation	Arctic mutation; E22G (= E693G)	Osaka mutation; E22Δ (= E693Δ)

### Organotypic hippocampal slice cultures

Organotypic hippocampal slice cultures were prepared from P6-P8 mice and cultured as previously described [13], with 3 slices per well. For assessment of dendritic spine density, cultures were transduced with Sindbis virus expressing EGFP at DIV 12 and were fixed at DIV15 with 4% paraformaldehyde in PBS containing 4% sucrose for 2h at 4°C. After washing with PBS, cultures were mounted with Hydromount (National diagnostics, Atlanta, Georgia) and coverslipped. For analysis of hTau-dependent toxicity, slices were transduced at DIV 12 with Sindbis virus expressing EGFP-coupled human 441 wild-type tau. At DIV 16 culture medium was harvested for cytotoxicity assays and lysates were prepared for western blot analyses. To determine the effect of recombinant Aβ on dendritic spines, slices were treated with 1 μM recombinant Aβ from DIV 8 until fixation on DIV 15. For measurement of cell toxicity, 1 μM recombinant Aβ was applied from DIV 12 until DIV 16. This concentration was chosen as it was previously shown that 1 μM of recombinant wt Aβ induces significant loss of dendritic spines and a human tau-dependent neuronal cell death in this experimental system [13].

### Recombinant Aβ

Recombinant Aβ was prepared as described previously [8, 9, 14]. For reconstitution, lyophilized Aβ was dissolved in 10 mM NaOH and sonicated for 1 min. Concentration was confirmed using Nanodrop (Thermo Scientific) and if necessary, adjusted to the concentration of 62.5 μM using 10 mM NaOH. Before addition to cell culture medium, the Aβ solution was mixed in neutralization buffer (40 mM HCl, 50mM H3PO4-NaOH pH 7.4, 10 mM NaCl) in a 4:1 ratio yielding a 50 μM Aβ solution at pH7.4 which is immediately added to the cell culture in a 1:50 ratio. For all steps, LoBind tubes (Eppendorf, Germany) were used.

### Analysis of dendritic spine density

Apical dendritic segments length in CA1 stratum radiatum were imaged using Leica SP2 confocal laserscanning microscope, equipped with 63x objective (NA: 1.2) and 488nm Argon laser. CA1 apical dendrites were chosen for their straighter appearance compared to CA3 dendrites, which allows the imaging of a single dendritic segment and a less error-prone counting of spine numbers per dendrite. Further, it has been shown that Aβ affects spine numbers in CA1 and CA3 apical dendrites to a similar extent [15]. Images were taken at a size of 30x30 μm (512x512 pixel, voxel size: 0.05813x0.05813x0.25 μm) with one clearly visible dendritic segment of 30–40 μm length. Image stacks were processed to maximum projections, regions of interest were cropped if applicable and dendritic spine density was determined as spine counts per μm dendrite using ImageJ.

### Assessment of cell death

To determine cytotoxicity of Aβ, culture medium was harvested on DIV 16, frozen in liquid nitrogen and stored at -80°C until further analysis with CytotoxGlo assay (Promega, Madison, USA), according to the manufacturer’s recommendations. For measurement of human EGFP-tau-dependent toxicity, CytotoxGlo signals were normalized to EGFP fluorescence using microplate reader Synergy HT.

### Westernblot analysis

On DIV 16 slices were harvested in RIPA buffer (50 mM Tris-HCl, 150 mM NaCl, 2 mM EDTA, 1% NP-40, 0.5% deoxycholate, and 0.1% SDS, pH 8.0) containing phosphatase inhibitor cocktails 1 and 2 (Sigma) and protease inhibitor cocktail (Roche) and centrifuged at 5000xg for 10 min at 4°C. The supernatant was collected, frozen in liquid nitrogen and stored at -80°C. Samples were mixed with loading buffer, resolved using 10–20% SDS PAGE and transferred to nitrocellulose membranes (Millipore). Immunoblotting was performed using primary antibodies Anti-GFP to detect total EGFP-coupled tau (1:1000, Roche), anti phospho-Tau AT8 (1:200, Pierce), anti GAPDH (1:5000, Biodesign) and HRP-conjugated secondary antibody (1:2000, Amersham). Bands were detected using the Supersignal Femto Maximum Sensitivity Substrate (Thermo) and imaged with Fujifilm Las-3000 assuring that no pixels were saturated. Band intensities were quantified with ImageJ and corrected for background.

### Statistics

Data are presented as mean ± standard deviation (SD). All datapoints (n-numbers) are plotted individually in each bar graph. Statistical analysis was performed with GraphPad Prism 7.01 software using Shapiro-Wilk test for normal distribution followed by one-way ANOVA with Tukey's multiple comparison test. Exact p-values, mean differences and significances for all experiments are listed in S1 Table. For dendritic spine analysis, n = 1 refers to one image containing one dendritic segment. For cell death assessment, n = 1 represents the analysis of supernatant of one cell culture well in which 3 hippocampal slices were cultured. For western blot analysis, n = 1 refers to the lysate of one well, in which 3 hippocampal slices were cultured and pooled for analysis.

## Results

### Reduced dendritic spine density in slice cultures from Tg2576 and arcAβ- but not E22ΔAβ transgenic mice

**Fig 1:**
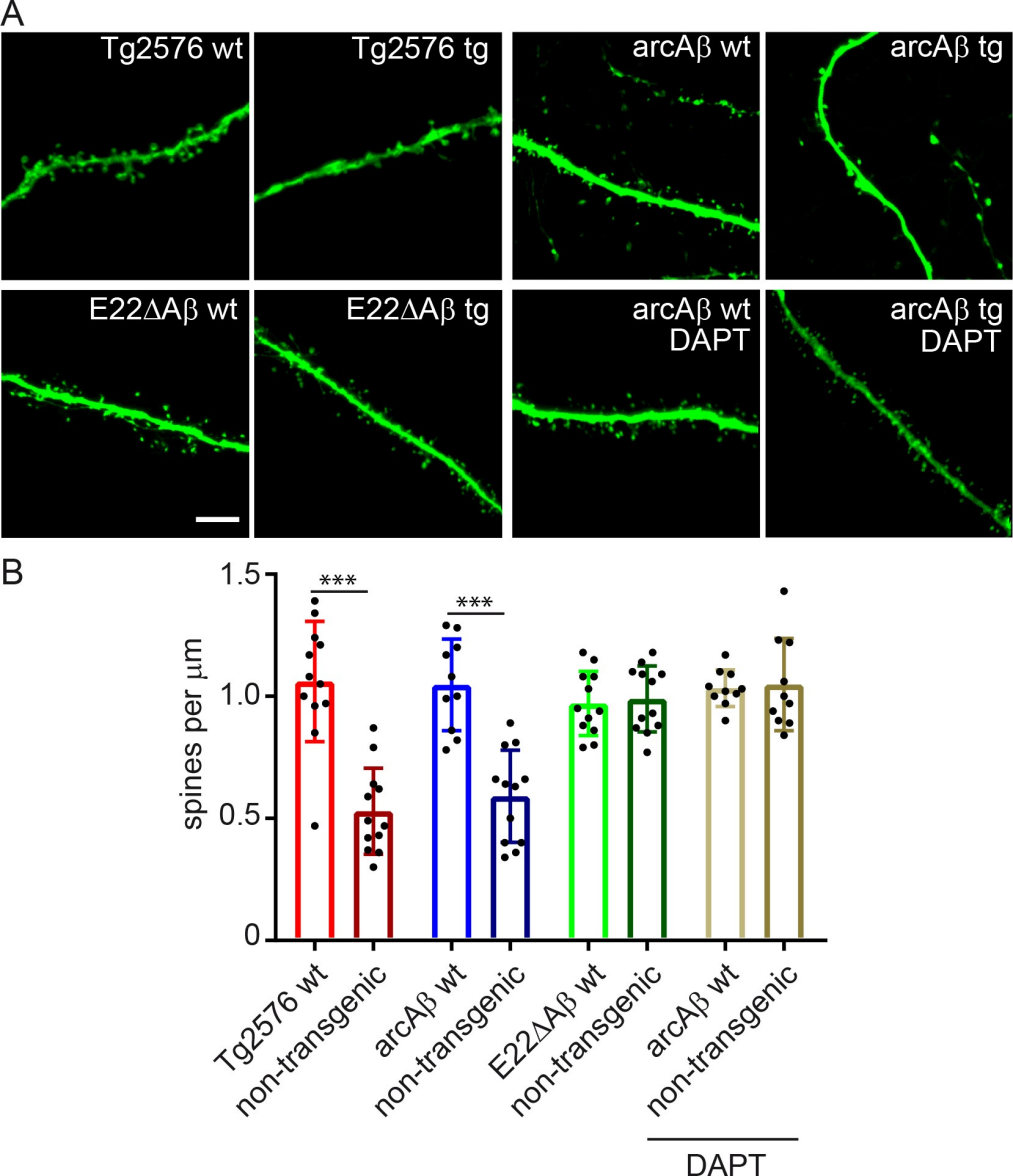
Reduction in dendritic spine density in hippocampal slices from Tg2576 and arcAβ- but not E22ΔAβ transgenic mice. A: Representative confocal images of dendritic segments of hippocampal slices from Tg2576, arcAβ- and E22ΔAβ transgenic mice and the respective non-transgenic littermates. B: Spine counts per μm dendrite. Spine density was reduced in cultures from Tg2576 tg mice and arcAβ tg mice but not in E22ΔAβ tg mice. Treatment with 0.5 μM DAPT abolished spine reduction in arcAβ tg slices. n = 10–12; Data are means ± SD. Statistical significance was assessed by one-way ANOVA with Tukey's multiple comparison test (***p<0.001). Scale bar: 5 μm.

Loss of pre- and postsynaptic structures is an early feature of AD and directly correlates to the degree of dementia [17]. Whether different intra-Aβ mutations confer a different degree of synaptotoxicity to Aβ is largely unknown. To determine the differential mechanisms of synapse loss caused by intra-Aβ mutants, we applied the well-established organotypic hippocampal slice culture technique [13, 15, 18, 19]. Slices were obtained from i) Tg2576 transgenic mice, which do not have an intra-Aβ mutation thus producing wild-type Aβ, ii) arcAβ transgenic mice, producing Aβ with the “Arctic” mutation E22G and iii) E22ΔAβ transgenic mice with the “Osaka” mutation E22Δ (Table 1). Slices were transduced with EGFP-expressing Sindbis virus, allowing high-resolution imaging of dendritic segments and spines [19]. As shown previously [13], spine density was strongly reduced in slices from arcAβ mice compared to slices from non-transgenic (non-tg) littermates (Fig 1A and 1B). This was rescued by treatment with the γ-secretase inhibitor DAPT at 0.5 μM indicating that spine reduction is mediated by Aβ and showing the validity of the transgenic slice culture model. Slices from Tg2576 mice displayed a similar reduction in spine numbers compared to arcAβ tg mice whereas E22ΔAβ tg slices showed no differences to their non-tg control (E22ΔAβ wt). A detailed list of all p-values is presented in S1 Table.

### Recombinant wt and E22G Aβ but not E22Δ Aβ reduce dendritic spine density

**Fig 2:**
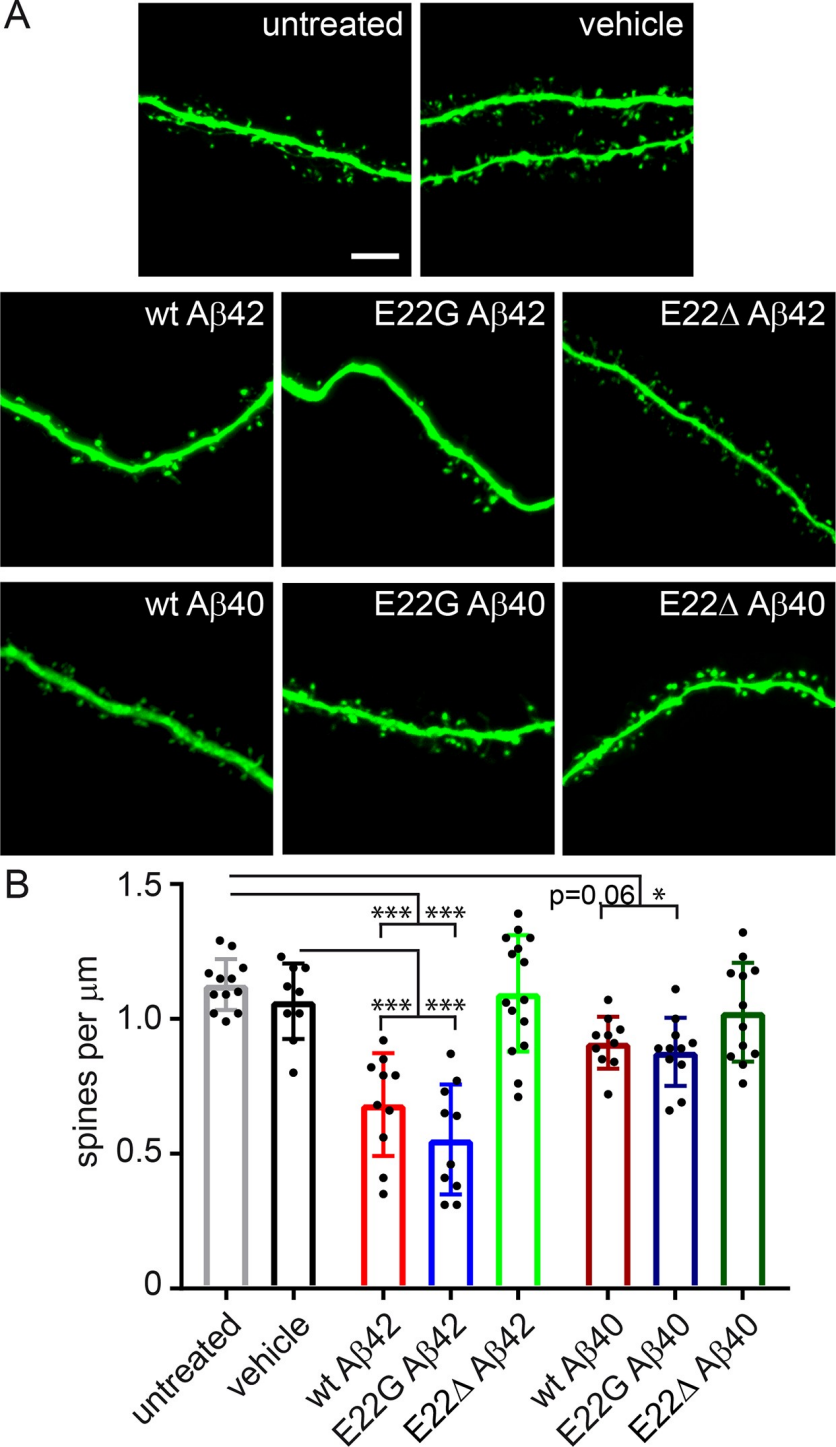
Reduction in dendritic spine density in hippocampal slices treated with recombinant wt and E22G Aβ but not E22Δ Aβ. A: Representative confocal images of dendritic segments of wild-type mice treated with 1 μM recombinant Aβ for 7 days. B: Spine counts per μm dendrite. Spine density was reduced after treatment with wt Aβ42 and E22G Aβ42 but not E22Δ Aβ42. Incubation with wt Aβ40 and E22G Aβ40 caused a mild reduction in spine numbers, whereas no effect was observed for E22Δ Aβ40. n = 10–15; Data are means ± SD. Statistical significance was assessed by one-way ANOVA with Tukey's multiple comparison test (*p<0.05; ***p<0.001). Scale bar: 5 μm.

To confirm the results obtained in slices from the transgenic mice, we used wild-type slices treated with recombinant Aβ containing the respective intra-Aβ mutations. Recombinant Aβ was produced in *Escherichia coli* with higher purity than commercially available synthetic Aβ [14]. Wt, E22G and E22Δ Aβ were tested at a concentration of 1 μM as 40- and 42-residue isoforms. Treatment with wt Aβ42 and E22G Aβ42 but not E22Δ Aβ42 induced a strong reduction in dendritic spine density compared to untreated and vehicle treated slices (Fig 2A and 2B). Interestingly, the degree of spine loss caused by wt Aβ42 and E22G Aβ42 was similar to the spine loss observed in slices from the respective transgenic mouse model (Fig 1). Wt Aβ40 and E22G Aβ40 caused spine loss to a lesser extent, with wt Aβ40 not reaching significance (p = 0.06). E22Δ Aβ40 did not show an effect on spine density. A direct comparison between the 40- and 42-residue isoforms showed that E22G Aβ42 reduced spine numbers significantly more than E22G Aβ40 (S1A Fig). While spine reduction upon wt Aβ42 treatment was similarly stronger than upon treatment with wt Aβ40 treatment, this difference did not reach significance (p = 0.059). However, no difference was observed between E22Δ Aβ42 and Aβ40 (p = 0.909) (S1A Fig) with both isoforms showing no significant effect on dendritic spines.

### Wt and E22G Aβ but not E22Δ Aβ induce of tau phosphorylation and tau-dependent cell death

**Fig 3:**
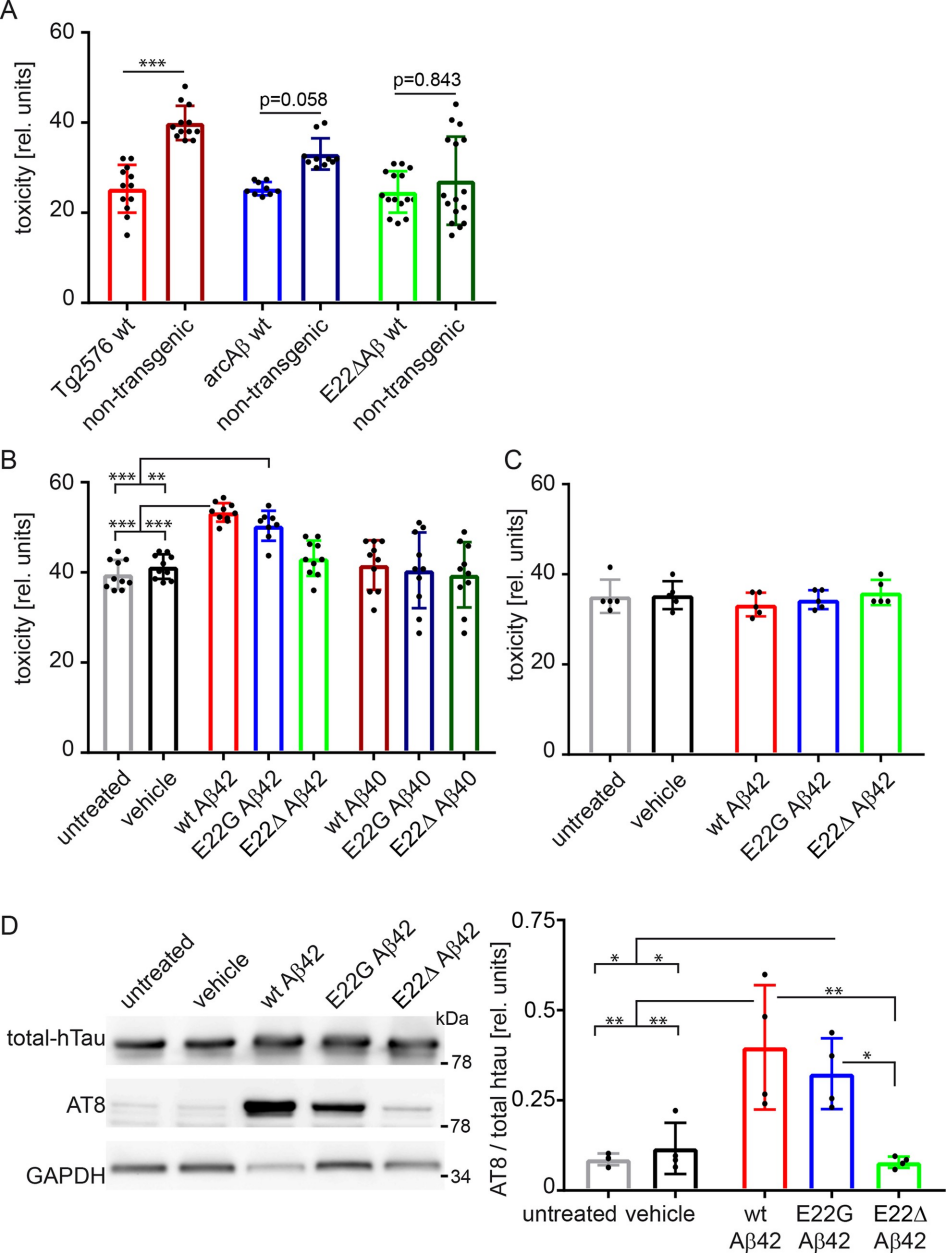
Induction of tau phosphorylation and tau-dependent cell death by wt and E22G Aβ but not E22Δ Aβ. A: Cytotoxicity in human tau expressing slices from Tg2576, arcAβ- and E22ΔAβ tg mice and the respective non-transgenic littermates, measured with CytotoxGlo assay. Increased cell death was observed in Tg2576 and arcAβ tg slices, although not significant in arcAβ tg slices, but not in E22ΔAβ tg slices. n = 9–16. B: Cytotoxicity in human tau expressing slices from wild-type mice treated with 1 μM recombinant Aβ. Treatment with wt Aβ42 and E22G Aβ42 but not E22Δ Aβ42, wt Aβ40, E22G Aβ40 or E22Δ Aβ40 increased cell death. n = 8–10 C: Cytotoxicity in slices from wild-type mice treated with 1 μM recombinant Aβ, in the absence of human tau expression. No toxicity was observed by wt Aβ42, E22G Aβ42 or E22Δ Aβ42 in the absence of human tau. n = 5. D: Representative western blot (left) of lysate of human tau expressing wild-type slices, treated with 1 μM recombinant Aβ, displaying total human tau, phospho-tau at the AT8 epitope and GAPDH. Quantification (right) of western blots showed increased tau phosphorylation after treatment with wt Aβ42 and E22G Aβ42 but not E22Δ Aβ42. n = 4. Data are means ± SD. Statistical significance was assessed by one-way ANOVA with Tukey's multiple comparison test (*p<0.05; **p<0.01, ***p<0.001).

We and others have shown that Aβ induces cell death, depending on the presence of tau [20], especially of human tau [15]. To determine whether intra-Aβ mutations confer different levels of toxicity to Aβ, we first used slices from Tg2576, arcAβ tg and E22ΔAβ tg mice, and virally expressed the 441 isoform of human tau, coupled to EGFP. Toxicity was determined using CytotoxGlo assay, measuring dead-cell protease activity released from cells that have lost membrane integrity (Fig 3A). A significantly higher toxicity was observed in slices from Tg2576 mice compared non-tg littermates. Higher signals were also found in arcAβ tg compared to non-tg slices, although this did not reach significance (p = 0.058). No difference was observed between slices from E22ΔAβ tg and non-tg littermates (p = 0.843).

To support this finding, slices from wild-type mice expressing human tau were treated with 1 μM recombinant Aβ (Fig 3B). Similar to the findings in transgenic cultures, wt Aβ42 and E22G Aβ42 treatment increased toxicity compared to untreated and vehicle treated slices. Wt Aβ40 and E22G Aβ40 did not induce toxicity. E22Δ Aβ42 and E22Δ Aβ40 both did not affect cell viability in this setting, confirming the results from E22ΔAβ tg cultures. A direct comparison between the 40- and 42-residue isoforms showed that wt Aβ42 and E22G Aβ42 were significantly more toxic than wt Aβ40 and E22G Aβ40, respectively, whereas no difference was observed between E22Δ Aβ42 and E22Δ Aβ40 (p = 0.688) (S1B Fig). To further confirm that Aβ toxicity is mediated by the presence of human tau, wild-type slices were treated with 1μM recombinant Aβ in the absence of human tau expression (Fig 3C). In this case, no Aβ toxicity was observed.

Is has been shown that Aβ can activate tau kinases thereby increasing tau phosphorylation which induces cell death downstream of Aβ [15, 21]. We determined the level of tau phosphorylation at the AT8 epitope, in cultures treated with wt Aβ42, E22G Aβ42 or E22Δ Aβ42 (Fig 3D). In line with our findings on cell toxicity, only wt Aβ42 and E22G Aβ42 but not E22Δ Aβ42 significantly increased tau phosphorylation.

Taken together, wt and E22G Aβ –either recombinant or transgenic–but not E22Δ Aβ increased tau phosphorylation and tau-dependent cell death.

## Discussion

In this study, we showed that the Osaka intra-Aβ mutation E693Δ did not confer toxic properties to Aβ in an ex vivo tissue culture model, compared to the Arctic intra-Aβ mutation E693G and to wt Aβ. We used either wild-type organotypic hippocampal slices treated with recombinant preparations of wt, E22G or E22Δ Aβ42 or Aβ40 or slices cultured from the respective transgenic mouse model, i.e. Tg2576, arcAβ and E22ΔAβ mice (Table 1). All mouse lines express APP_swe_ under the control of the prion protein promoter and differ only in the intra-Aβ mutation at position 22. Expression levels of APP are identical in these mice [8]. Several studies on E22Δ aggregation or toxicity used synthetic Aβ peptides. However, synthetically produced Aβ is often contaminated with up to 9% impurities, such as racemized peptides, that cannot be avoided due to the technical limitations of peptide synthesis [9, 14]. Importantly, impurities in synthetic Aβ already in the range of 3% were previously shown to have a significant impact on its aggregation properties [9, 14]. The use of recombinant Aβ can bypass this limitation and it has been shown that recombinantly produced Aβ aggregates faster and is more toxic than synthetic Aβ [14]. In a previous study, we compared the aggregation propensity of recombinant wt, E22G and E22Δ Aβ42 and Aβ40 and found unique aggregation characteristics of E22Δ Aβ42 and Aβ40 [8]. Compared to E22G and wt Aβ, E22Δ Aβ42 displayed highest fibrillogenesis propensity as it aggregated without a lag phase in a thioflavin T assay and formed SDS-stable high molecular weight aggregates directly after reconstitution whereas fibril growth after reconstitution occurred slowly and non-exponentially. Thus, addition of recombinant E22Δ Aβ to our slice cultures may have caused rapid formation of less toxic fibrils in the extracellular space, which may explain the lack of toxicity in this setting (see also discussion below).

Loss of pre- and postsynaptic contacts is a major correlate to the degree of dementia in AD [17]. We showed that recombinant wt Aβ42 and E22G Aβ42 strongly reduced the numbers of dendritic spines in slice cultures while wt Aβ40 and E22G Aβ40 only had a mild effect. As Aβ treatment in the absence of human tau expression is not toxic in our model, the reduction in dendritic spine density represents a distinct pathophysiological event caused by Aβ rather than a general toxic effect. Importantly, the degree of spine loss in the Aβ42-treated wild-type slices was similar to spine loss in slices from the respective transgenic mice, confirming that the treatment with recombinant Aβ can mirror the effects of Aβ produced from processing of APP. Further, the protective effect of γ-secretase inhibitor DAPT indicates that the synaptotoxic events in these transgenic cultures were indeed caused by Aβ. In contrast to wt and E22G Aβ, dendritic spine loss was neither observed in wild-type slices treated with recombinant E22Δ Aβ42 or Aβ40 nor in slices from E22ΔAβ transgenic mice. This is opposed to a study which showed that synthetic E22Δ Aβ caused loss of synaptophysin signals in stained slice cultures [22], although treatment with 1 μM synthetic Aβ42 did not cause a significant reduction in the synaptophysin signal in that study. Further, primary cultures transfected with E693Δ APP showed a loss of dendritic spine numbers compared to wt APP transfected cells [23]. These discrepancies to our data may be explained by the different techniques used. As discussed above, synthetic and recombinant Aβ have different aggregation kinetics probably leading to different types of aggregates. Further, lipofected primary neurons strongly differ from transgenic slice cultures due do differential expression levels, time-course of expression and different properties of tissue and dissociated cultures (see below). Third, our transgenic models bear the APP_swe_ mutation in addition to the intra-Aβ mutation, which increases total Aβ production. Nevertheless, we did not observe any effect on spine density in cultures from E22ΔAβ transgenic mice. In addition to the number of spines, spine morphology has been shown to be affected in AD models [24]. Primary neurons transfected with E693Δ APP displayed a specific loss of mushroom-shaped spines, but not of stubby or thin spines [23]. A loss of mushroom spines was also observed in APP_SDL_ transgenic slice cultures, which produce Aβ with the E693Q (Dutch) mutation [15, 25], as well as in hippocampal neurons treated with wt Aβ oligomers [26]. However, both studies also showed that loss of mushroom spines was accompanied by an increase in the fraction of stubby spines, indicating a transition from mushroom to stubby shape rather than a specific loss of mushroom spines. Nevertheless, a direct comparison of the different Aβ mutants and their effect on spine morphology in the same experimental system should be performed in the future.

Beside the loss of synapses, massive neurodegeneration is characteristic for AD. Previous studies from our lab showed that both transgenically produced Aβ and treatment with recombinant wt Aβ induced neurodegeneration in slice cultures, which was prominent in the presence of human tau expression [13, 15]. Here, we showed that wt and E22G Aβ induced a tau-dependent cell death. As tau was expressed using neurotrophic Sindbis virus, we conclude that the affected cells were primarily neurons. In contrast, E22Δ Aβ did not show induction of toxicity. The absence of neurotoxocity is likely due to the the lack of induced tau phosphorylation by E22Δ Aβ. While wt Aβ42 and E22G Aβ42 massively increases AT8 phosphorylation of tau, treatment with E22Δ Aβ42 did not elevate phospho-tau signals above baseline. The AT8 epitope (Ser202/The205) is a major phosphorylation site in AD and used to classify the degree of AD pathology into different Braak stages [27]. Increased phosphorylation, among others at the AT8 epitope, has been shown to confer toxic properties to tau [13, 15]. Our data is in line with studies from other labs in which synthetic wt Aβ42 caused a strong MTT reduction in N2a and IMR-32 cells, while E22Δ Aβ42 only caused a slight reduction in MTT signal [22]. Further, treatment of primary rat cortical cultures with synthetic wt and E22P Aβ42 but not E22Δ Aβ40 or Aβ42 reduced cell survival [28]. Ovchinnikova and colleagues compared the toxicity of recombinant wt and E22Δ Aβ40 and Aβ42 [9]. When added to primary neuronal cultures wt Aβ42 strongly induced LDH release, while E22Δ Aβ42 did not affect cell survival. Interestingly, E22Δ Aβ40 showed a detectable cell toxicity, although not as strong as wt Aβ42. The reason why we did not observe cell toxicity of E22Δ Aβ40 may be explained by the use of a lower concentration (1 μM compared to 7.4 μM) and the use of an ex vivo tissue culture model. From our experience primary dissociated neuronal cultures are generally more susceptible to toxic insults than tissue cultures. In contrast to the lack of tau phosphorylation and tau-dependent cell death in our study, Umeda and colleagues found exacerbated tau pathology in APP_osk_-hTau double transgenic mice [29], suggesting that *in vivo* E22Δ Aβ may well affect tau biology. However, the study did not compare APP_osk_-hTau transgenic mice to APP_wt_-hTau transgenic mice or APP_arc_-hTau transgenic mice. Although speculative, APP_wt_-hTau or APP_arc_-hTau mice may have shown a stronger tau pathology than APP_osk_-hTau.

Taken together, we observed synaptic loss, tau phosphorylation and neuronal degeneration induced by wt and E22G Aβ, both in a transgenic model and in a recombinant form, while E22Δ Aβ did not display any effects. Nevertheless, the lack of toxicity of E22Δ Aβ in our model requires further explanation. We showed differential aggregation properties of recombinant E22Δ Aβ in our previous study [8]. The type of E22Δ Aβ oligomers which cause neuronal degeneration and synaptic loss in AD patients with the Osaka APP mutations my either not be present in our model (see also discussion above on aggregation propensity of recombinant E22Δ Aβ) or it may appear in the wrong localization. It has been speculated that E22Δ Aβ oligomers predominantly accumulate intra-neuronally and exert their toxic effects from inside the cell [30]. In line, Kondo and colleagues found intracellular Aβ oligomers in induced pluripotent stem cell-derived neurons from E693Δ AD patients but not in patients with the V717F APP mutation [31]. They hypothesized, that the pathophysiologic heterogeneity in AD may be due to the differential presence of intracellular Aβ oligomers. Hence, familial AD caused by E693Δ would belong to the intraneuronal-type of AD. E22ΔAβ tg mice, used in our study, display intraneuronal Aβ aggregates, associated with cognitive deficits, at 3 months of age [8]. However, as our slice cultures were prepared from P6-8 mice and cultured for 2 weeks, we do not expect the presence of intracellular Aβ accumulation, which may explain the lack of toxicity in this model. Interestingly, arcAβ (E22G) transgenic mice also accumulate intraneuronal Aβ, although at a later time point than E22ΔAβ tg mice. Nevertheless, we previously showed that wt and E22G Aβ toxicity depends on NMDA receptor signalling and that treatment of arcAβ transgenic cultures with anti-Aβ antibodies rescued Aβ toxicity, clearly indicating that both wt and E22G Aβ at least have an extracellular phase in their toxicity mechanisms [13, 15]. Collectively, our findings therefore support the hypothesis of an intraneuronal toxicity mechanism by E22ΔAβ.

### Pitfalls/limitations

We are aware that the main conclusion of our study, that E22G and E22Δ Aβ work via different mechanisms caused by differential aggregation properties and potential different localization of the aggregates, is only shown indirectly. A direct measure would be the analysis of Aβ aggregates and their localization in the slice culture model. However, our study was affected by a limited access to animals and tissue, that did not allow for further analysis Aβ aggregates or localization. Nevertheless, we base our conclusions on a combination of data obtained in the present study and of data from previous publications, where we showed differential properties and aggregation kinetics of wt, E22G and E22Δ Aβ in vitro and in vivo [8]. Thus, we are convinced that the conclusion drawn here are valid, however with taking the above-mentioned limitations into account.

## Supporting information

S1 Table P-values, mean differences and significances for all experiments. (PDF)

S1 Fig Comparison of spine loss and cell toxicity between the 40- and 42-residue variants of recombinant wt, E22G and E22Δ Aβ. These graphs summarize the findings of Figs 2B and 3B and were plotted to better compare the effects of wt Aβ42, E22G Aβ42 and E22Δ Aβ42 to the respective 40-residue variant, wt Aβ40, E22G Aβ40 and E22Δ Aβ40. A: Spine counts per μm dendrite. Spine density reduction after treatment with wt Aβ42 and E22G Aβ42 was higher compared to wt Aβ40 and E22G Aβ40, respectively. No difference was observed between E22Δ Aβ42 and E22Δ Aβ40. n = 10–15 B: Cytotoxicity in human tau-expressing slices from wild-type mice treated with 1 μM recombinant Aβ. Wt Aβ42 and E22Δ Aβ42 induced more cytotoxicity than wt Aβ40 and E22G Aβ40. No difference was observed between E22Δ Aβ42 and E22Δ s1Aβ40. n = 8–10. Data are means ± SD. Statistical significance was assessed by one-way ANOVA with Tukey's multiple comparison test (**p<0.01, ***p<0.001).
(TIF)

S1 File (PDF)
