# Supplementary material for: Familial Alzheimer’s disease mutations at position 22 of the amyloid β-peptide sequence differentially affect synaptic loss, tau phosphorylation and neuronal cell death in an ex vivo system
Source: PLoS One. 2020 Sep 23;15(9):e0239584. doi: 10.1371/journal.pone.0239584 (PMC7510992; doi:10.1371/journal.pone.0239584)
Supplement: S1 Table — (PDF) [file pone.0239584.s001.pdf]

RAW Data for Figure 1:

Dendtitic spine density:

Tg2576-wt	non-tg	ArcAβ-wt	non-tg	E22ΔAβ-wt	non-tg	ArcAβ-wt +DAPT	non-tg +DAPT
1	0.42	0.78	0.5	1.08	1.18	0.97	0.9
1.05	0.43	0.86	0.4	0.95	1.09	0.9	0.84
0.97	0.3	0.82	0.34	1.15	1.06	1.02	1.22
1.08	0.49	1.2	0.36	1.18	1.14	1.03	1.43
0.47	0.36	1	0.4	1.08	0.88	1	0.89
1.39	0.64	1.28	0.63	1.03	1.1	1.17	1.23
1.34	0.62	0.99	0.64	0.91	1.09	1.01	1.06
0.85	0.37	1.17	0.66	0.88	0.85	1.1	0.97
1.17	0.59	1.29	0.89	0.8	0.93	1.09	1
0.96	0.47	1.08	0.8	0.86	0.87	1.04	0.94
1.21	0.87		0.81	0.94	0.91		
1.24	0.79		0.66	0.79	0.77		

Statistical analysis for Figure 1:
one-way ANOVA with Tukey's multiple comparison test
GraphPad Prism 7.01

Sample comparison	Mean Diff.	Significant?	Summary	Adjusted P Value
Tg2576-non-tg vs. Tg2576-tg	0.532	Yes	****	<0.0001
Tg2576-non-tg vs. ArcAβ-non-tg	0.0138	No	ns	>0.9999
Tg2576-non-tg vs. ArcAβ-tg	0.47	Yes	****	<0.0001
Tg2576-non-tg vs. E22ΔAβ-non-tg	0.09	No	ns	0.9079
Tg2576-non-tg vs. E22ΔAβ-tg	0.0717	No	ns	0.9717
Tg2576-non-tg vs. ArcAβ-non-tg + DAPT	0.0278	No	ns	>0.9999
Tg2576-non-tg vs. ArcAβ-tg + DAPT	0.0128	No	ns	>0.9999
Tg2576-tg vs. ArcAβ-non-tg	-0.518	Yes	****	<0.0001
Tg2576-tg vs. ArcAβ-tg	-0.0617	No	ns	0.988
Tg2576-tg vs. E22ΔAβ-non-tg	-0.442	Yes	****	<0.0001
Tg2576-tg vs. E22ΔAβ-tg	-0.46	Yes	****	<0.0001
Tg2576-tg vs. ArcAβ-non-tg + DAPT	-0.504	Yes	****	<0.0001
Tg2576-tg vs. ArcAβ-tg + DAPT	-0.519	Yes	****	<0.0001
ArcAβ-non-tg vs. ArcAβ-tg	0.456	Yes	****	<0.0001
ArcAβ-non-tg vs. E22ΔAβ-non-tg	0.0762	No	ns	0.9696
ArcAβ-non-tg vs. E22ΔAβ-tg	0.0578	No	ns	0.9939
ArcAβ-non-tg vs. ArcAβ-non-tg + DAPT	0.014	No	ns	>0.9999
ArcAβ-non-tg vs. ArcAβ-tg + DAPT	-0.001	No	ns	>0.9999
ArcAβ-tg vs. E22ΔAβ-non-tg	-0.38	Yes	****	<0.0001
ArcAβ-tg vs. E22ΔAβ-tg	-0.398	Yes	****	<0.0001
ArcAβ-tg vs. ArcAβ-non-tg + DAPT	-0.442	Yes	****	<0.0001
ArcAβ-tg vs. ArcAβ-tg + DAPT	-0.457	Yes	****	<0.0001
E22ΔAβ-non-tg vs. E22ΔAβ-tg	-0.0183	No	ns	>0.9999
E22ΔAβ-non-tg vs. ArcAβ-non-tg + DAPT	-0.0622	No	ns	0.9905
E22ΔAβ-non-tg vs. ArcAβ-tg + DAPT	-0.0772	No	ns	0.9673
E22ΔAβ-tg vs. ArcAβ-non-tg + DAPT	-0.0438	No	ns	0.9989
E22ΔAβ-tg vs. ArcAβ-tg + DAPT	-0.0588	No	ns	0.9932
ArcAβ-non-tg + DAPT vs. ArcAβ-tg + DAPT	-0.015	No	ns	>0.9999

Tg2576-non-tg refers to the non-tragengenic littermates of the Tg2576-tg mice.
ArcAβ-non-tg refers to the non-tragengenic littermates of the ArcAβ-tg mice.
E22ΔAβ-non-tg refers to the non-tragengenic littermates of the E22ΔAβ-tg mice.

RAW Data for Figure 2:

Dendtitic spine density:

untreated	vehicle	wt Aβ42	E22G Aβ42	E22Δ Aβ42	wt Aβ40	E22G Aβ40	E22Δ Aβ42
1.27	0.8	0.66	0.38	1.3	0.72	0.89	1.23
1.02	1.23	0.41	0.41	0.71	0.85	0.85	0.83
1.29	1.19	0.79	0.64	1.07	0.94	0.89	0.97
1.09	1.1	0.92	0.77	0.99	1	0.89	1.05
1.15	1.12	0.56	0.65	1.26	0.84	0.83	0.76
1.1	0.92	0.35	0.46	1.03	0.94	1.11	1.32
1.19	1.02	0.82	0.31	1.24	0.96	0.94	0.86
1.15	1.19	0.68	0.73	1.3	0.91	0.91	0.9
1.02	1.02	0.79	0.31	1.21	0.89	0.66	1.16
0.99		0.85	0.87	0.88	1.07	0.69	0.87
1.17				1.33		1	1.18
1.09				1.39			1.17
				1.06			
				0.9			
				0.76			

Statistical analysis for Figure 2:
one-way ANOVA with Tukey's multiple comparison test
GraphPad Prism 7.01

Sample comparison	Mean Diff.	Significant?	Summary	Adjusted P Value
untreated vs. vehicle	0.0619	No	ns	0.9896
untreated vs. wt Aβ42	0.445	Yes	****	<0.0001
untreated vs. E22GAβ42	0.575	Yes	****	<0.0001
untreated vs. E22ΔAβ42	0.0322	No	ns	0.9996
untreated vs. wt 40	0.216	No	ns	0.0604
untreated vs. E22G40	0.249	Yes	*	0.0119
untreated vs. E22Δ40	0.103	No	ns	0.7964
vehicle vs. wt Aβ42	0.383	Yes	****	<0.0001
vehicle vs. E22GAβ42	0.513	Yes	****	<0.0001
vehicle vs. E22ΔAβ42	-0.0298	No	ns	0.9999
vehicle vs. wt 40	0.154	No	ns	0.4762
vehicle vs. E22G40	0.187	No	ns	0.203
vehicle vs. E22Δ40	0.0406	No	ns	0.9993
wt Aβ42 vs. E22GAβ42	0.13	No	ns	0.6512
wt Aβ42 vs. E22ΔAβ42	-0.412	Yes	****	<0.0001
wt Aβ42 vs. wt 40	-0.229	No	ns	0.0526
wt Aβ42 vs. E22G40	-0.195	No	ns	0.1382
wt Aβ42 vs. E22Δ40	-0.342	Yes	***	0.0002
E22GAβ42 vs. E22ΔAβ42	-0.542	Yes	****	<0.0001
E22GAβ42 vs. wt 40	-0.359	Yes	***	0.0002
E22GAβ42 vs. E22G40	-0.325	Yes	***	0.0006
E22GAβ42 vs. E22Δ40	-0.472	Yes	****	<0.0001
E22ΔAβ42 vs. wt 40	0.183	No	ns	0.1338
E22ΔAβ42 vs. E22G40	0.217	Yes	*	0.0294
E22ΔAβ42 vs. E22Δ40	0.0703	No	ns	0.9557
wt 40 vs. E22G40	0.0338	No	ns	0.9998
wt 40 vs. E22Δ40	-0.113	No	ns	0.7525
E22G40 vs. E22Δ40	-0.147	No	ns	0.4086

RAW Data for Figure 3:

A:

Cytotoy Assay (rel. Units):

Tg2576-wt	non-tg	ArcAβ-wt	non-tg	E22ΔAβ-wt	non-tg
21.00	45.00	27.36	30.62	29.98	36.16
23.00	48.00	27.36	39.91	30.87	40.57
15.00	38.00	26.71	29.96	28.22	36.16
19.00	39.00	24.75	31.27	22.93	20.28
26.00	36.00	24.75	31.27	23.81	22.05
28.00	38.00	25.40	31.92	22.93	21.17
23.00	40.00	24.10	31.92	17.64	14.99
26.00	38.00	23.45	32.57	18.52	17.64
30.00	44.00	24.10	31.92	30.87	35.28
32.00	40.00		39.08	29.10	44.10
29.00	36.00			28.22	39.69
32.00	37.00			22.05	24.69
				22.93	22.93
				22.93	23.81
				18.52	16.76
					17.64

B:

Cytotoy Assay:

untreated	vehicle	wt Aβ42	E22G Aβ42	E22Δ Aβ42	wt Aβ40	E22G Aβ40	E22Δ Aβ42
18.20	20.01	26.20	26.36	20.32	23.46	24.99	14.79
19.32	19.20	27.11	25.92	18.00	23.46	17.34	13.26
22.33	21.69	26.79	24.00	22.96	21.93	25.50	20.40
21.05	22.33	27.43	21.85	19.77	23.46	24.48	18.36
18.43	19.28	27.85	25.28	21.00	15.81	20.40	19.89
18.00	18.85	28.28	25.71	21.43	17.85	21.93	18.87
18.85	21.00	25.71	27.42	24.00	22.44	20.40	20.91
19.28	20.14	24.85	26.14	21.43	21.42	19.38	22.95
21.43	22.28	26.14		24.00	20.40	13.26	23.46
21.43	21.85	26.57		23.14	17.85	14.79	24.48

C:

Cytotox Assay:

untreated	vehicle	wt Aβ42	E22G Aβ42	E22Δ Aβ42
16.0	18.0	18.0	17.0	17.0
17.0	20.0	18.0	16.0	20.0
20.8	17.6	15.1	16.4	17.0
17.0	15.8	16.4	18.3	17.0
17.0	17.0	15.8	18.3	18.9

D:

Western blot (rel. Units):

untreated	vehicle	wt Aβ42	E22G Aβ42	E22Δ Aβ42
0.10252457	0.09637935	0.5991973	0.3733438	0.09514805
0.08933795	0.22133976	0.24085441	0.25506339	0.08536898
0.09015963	0.0845228	0.48206893	0.43752766	0.07526084
0.06408804	0.06545653	0.26741373	0.23066941	0.05850462

Statistical analysis for Figure 3:
one-way ANOVA with Tukey's multiple comparison test
GraphPad Prism 7.01

A:

Sample comparison	Mean Diff.	Significant?	Summary	Adjusted P Value
Tg2576-non-tg vs. Tg2576-tg	-14.6	Yes	****	<0.0001
Tg2576-non-tg vs. ArcAβ-non-tg	0.00111	No	ns	>0.9999
Tg2576-non-tg vs. ArcAβ-tg	-7.71	Yes	*	0.0339
Tg2576-non-tg vs. E22Δ-non-tg	0.699	No	ns	0.9996
Tg2576-non-tg vs. E22Δ-tg	-1.79	No	ns	0.9666
Tg2576-tg vs. ArcAβ-non-tg	14.6	Yes	****	<0.0001
Tg2576-tg vs. ArcAβ-tg	6.87	No	ns	0.0792
Tg2576-tg vs. E22Δ-non-tg	15.3	Yes	****	<0.0001
Tg2576-tg vs. E22Δ-tg	12.8	Yes	****	<0.0001
ArcAβ-non-tg vs. ArcAβ-tg	-7.71	No	ns	0.0583
ArcAβ-non-tg vs. E22Δ-non-tg	0.698	No	ns	0.9997
ArcAβ-non-tg vs. E22Δ-tg	-1.79	No	ns	0.977
ArcAβ-tg vs. E22Δ-non-tg	8.41	Yes	**	0.0095
ArcAβ-tg vs. E22Δ-tg	5.93	No	ns	0.1341
E22Δ-non-tg vs. E22Δ-tg	-2.48	No	ns	0.8436

Tg2576-non-tg refers to the non-tragengenic littermates of the Tg2576-tg mice.
ArcAβ-non-tg refers to the non-tragengenic littermates of the ArcAβ-tg mice.
E22ΔAβ-non-tg refers to the non-tragengenic littermates of the E22ΔAβ-tg mice.

B:

Sample comparison	Mean Diff.	Significant?	Summary	Adjusted P Value
untreated vs. vehicle	-0.831	No	ns	0.9956
untreated vs. wt Aβ42	-6.86	Yes	****	<0.0001
untreated vs. E22GAβ42	-5.5	Yes	***	0.0005
untreated vs. E22ΔAβ42	-1.77	No	ns	0.7666
untreated vs. wt Aβ40	-0.976	No	ns	0.9883
untreated vs. E22GAβ40	-0.415	No	ns	>0.9999
untreated vs. E22ΔAβ40	0.095	No	ns	>0.9999
vehicle vs. wt Aβ42	-6.03	Yes	****	<0.0001
vehicle vs. E22GAβ42	-4.67	Yes	**	0.0051
vehicle vs. E22ΔAβ42	-0.942	No	ns	0.9905
vehicle vs. wt Aβ40	-0.145	No	ns	>0.9999
vehicle vs. E22GAβ40	0.416	No	ns	>0.9999
vehicle vs. E22ΔAβ40	0.926	No	ns	0.9914
wt Aβ42 vs. E22GAβ42	1.36	No	ns	0.9471
wt Aβ42 vs. E22ΔAβ42	5.09	Yes	***	0.0007
wt Aβ42 vs. wt Aβ40	5.89	Yes	****	<0.0001
wt Aβ42 vs. E22GAβ40	6.45	Yes	****	<0.0001
wt Aβ42 vs. E22ΔAβ40	6.96	Yes	****	<0.0001
E22GAβ42 vs. E22ΔAβ42	3.73	No	ns	0.0516
E22GAβ42 vs. wt Aβ40	4.53	Yes	**	0.0075
E22GAβ42 vs. E22GAβ40	5.09	Yes	**	0.0016
E22GAβ42 vs. E22ΔAβ40	5.6	Yes	***	0.0004
E22ΔAβ42 vs. wt Aβ40	0.797	No	ns	0.9966
E22ΔAβ42 vs. E22GAβ40	1.36	No	ns	0.9287
E22ΔAβ42 vs. E22ΔAβ40	1.87	No	ns	0.7164
wt Aβ40 vs. E22GAβ40	0.561	No	ns	0.9996
wt Aβ40 vs. E22ΔAβ40	1.07	No	ns	0.98
E22GAβ40 vs. E22ΔAβ40	0.51	No	ns	0.9998

C:

Sample comparison	Mean Diff.	Significant?	Summary	Adjusted P Value
untreated vs. vehicle	-0.118	No	ns	>0.9999
untreated vs. wt Aβ42	0.912	No	ns	0.8572
untreated vs. E22GAβ42	0.378	No	ns	0.9936
untreated vs. E22ΔAβ42	-0.422	No	ns	0.9903
vehicle vs. wt Aβ42	1.03	No	ns	0.7959
vehicle vs. E22GAβ42	0.496	No	ns	0.9822
vehicle vs. E22ΔAβ42	-0.304	No	ns	0.9972
wt Aβ42 vs. E22GAβ42	-0.534	No	ns	0.9767
wt Aβ42 vs. E22ΔAβ42	-1.33	No	ns	0.6065
E22GAβ42 vs. E22ΔAβ42	-0.8	No	ns	0.9054

D:

Sample comparison	Mean Diff.	Significant?	Summary	Adjusted P Value
untreated vs. vehicle	-0.0304	No	ns	0.9903
untreated vs. wt Aβ42	-0.311	Yes	**	0.0025
untreated vs. E22GAβ42	-0.238	Yes	*	0.0209
untreated vs. E22ΔAβ42	0.00796	No	ns	>0.9999
vehicle vs. wt Aβ42	-0.28	Yes	**	0.0061
vehicle vs. E22GAβ42	-0.207	Yes	*	0.0495
vehicle vs. E22ΔAβ42	0.0384	No	ns	0.9772
wt Aβ42 vs. E22GAβ42	0.0732	No	ns	0.8073
wt Aβ42 vs. E22ΔAβ42	0.319	Yes	**	0.002
E22GAβ42 vs. E22ΔAβ42	0.246	Yes	*	0.0166

RAW Data for S1Figure:

A:

Dendtitic spine density:

wt Aβ42	wt Aβ40	E22G Aβ42	E22G Aβ40	E22Δ Aβ42	E22Δ Aβ42
0.66	0.72	0.38	0.89	1.3	1.23
0.41	0.85	0.41	0.85	0.71	0.83
0.79	0.94	0.64	0.89	1.07	0.97
0.92	1	0.77	0.89	0.99	1.05
0.56	0.84	0.65	0.83	1.26	0.76
0.35	0.94	0.46	1.11	1.03	1.32
0.82	0.96	0.31	0.94	1.24	0.86
0.68	0.91	0.73	0.91	1.3	0.9
0.79	0.89	0.31	0.66	1.21	1.16
0.85	1.07	0.87	0.69	0.88	0.87
			1	1.33	1.18
				1.39	1.17
				1.06	
				0.9	
				0.76	

B:

Cytotox Assay

wt Aβ42	wt Aβ40	E22G Aβ42	E22G Aβ40	E22Δ Aβ42	E22Δ Aβ42
26.2	23.46	26.36	24.99	20.32	14.79
27.11	23.46	25.92	17.34	18	13.26
26.79	21.93	24	25.5	22.96	20.4
27.43	23.46	21.85	24.48	19.77	18.36
27.85	15.81	25.28	20.4	21	19.89
28.28	17.85	25.71	21.93	21.43	18.87
25.71	22.44	27.42	20.4	24	20.91
24.85	21.42	26.14	19.38	21.43	22.95
26.14	20.4		13.26	24	23.46
26.57	17.85		14.79	23.14	24.48

Statistical analysis for S1 Figure:
one-way ANOVA with Tukey's multiple comparison test
GraphPad Prism 7.01

A:

Sample comparison	Mean Diff.	Significant?	Summary	Adjusted P Value
wt Aβ42 vs. wt Aβ40	-0.229	No	ns	0.0586
wt Aβ42 vs. E22GAβ42	0.13	No	ns	0.58
wt Aβ42 vs. E22GAβ40	-0.1952	No	ns	0.1369
wt Aβ42 vs. E22ΔAβ42	-0.4123	Yes	****	<0.0001
wt Aβ42 vs. E22ΔAβ40	-0.342	Yes	***	0.0004
wt Aβ40 vs. E22GAβ42	0.359	Yes	***	0.0004
wt Aβ40 vs. E22GAβ40	0.03382	No	ns	0.9979
wt Aβ40 vs. E22ΔAβ42	-0.1833	No	ns	0.1331
wt Aβ40 vs. E22ΔAβ40	-0.113	No	ns	0.6762
E22GAβ42 vs. E22GAβ40	-0.3252	Yes	**	0.0012
E22GAβ42 vs. E22ΔAβ42	-0.5423	Yes	****	<0.0001
E22GAβ42 vs. E22ΔAβ40	-0.472	Yes	****	<0.0001
E22GAβ40 vs. E22ΔAβ42	-0.2172	Yes	*	0.0354
E22GAβ40 vs. E22ΔAβ40	-0.1468	No	ns	0.3674
E22ΔAβ42 vs. E22ΔAβ40	0.07033	No	ns	0.9093

B:

Sample comparison	Mean Diff.	Significant?	Summary	Adjusted P Value
wt Aβ42 vs. wt Aβ40	5.885	Yes	***	0.0003
wt Aβ42 vs. E22GAβ42	1.358	No	ns	0.9086
wt Aβ42 vs. E22GAβ40	6.446	Yes	****	<0.0001
wt Aβ42 vs. E22ΔAβ42	5.088	Yes	**	0.0022
wt Aβ42 vs. E22ΔAβ40	6.956	Yes	****	<0.0001
wt Aβ40 vs. E22GAβ42	-4.527	Yes	*	0.0153
wt Aβ40 vs. E22GAβ40	0.561	No	ns	0.9976
wt Aβ40 vs. E22ΔAβ42	-0.797	No	ns	0.9877
wt Aβ40 vs. E22ΔAβ40	1.071	No	ns	0.9554
E22GAβ42 vs. E22GAβ40	5.088	Yes	**	0.0045
E22GAβ42 vs. E22ΔAβ42	3.73	No	ns	0.0724
E22GAβ42 vs. E22ΔAβ40	5.598	Yes	**	0.0014
E22GAβ40 vs. E22ΔAβ42	-1.358	No	ns	0.8857
E22GAβ40 vs. E22ΔAβ40	0.51	No	ns	0.9985
E22ΔAβ42 vs. E22ΔAβ40	1.868	No	ns	0.6717
