# Supplementary figures and images for: Familial Alzheimer’s disease mutations at position 22 of the amyloid β-peptide sequence differentially affect synaptic loss, tau phosphorylation and neuronal cell death in an ex vivo system
Source: PLoS One. 2020 Sep 23;15(9):e0239584. doi: 10.1371/journal.pone.0239584 (PMC7510992; doi:10.1371/journal.pone.0239584)

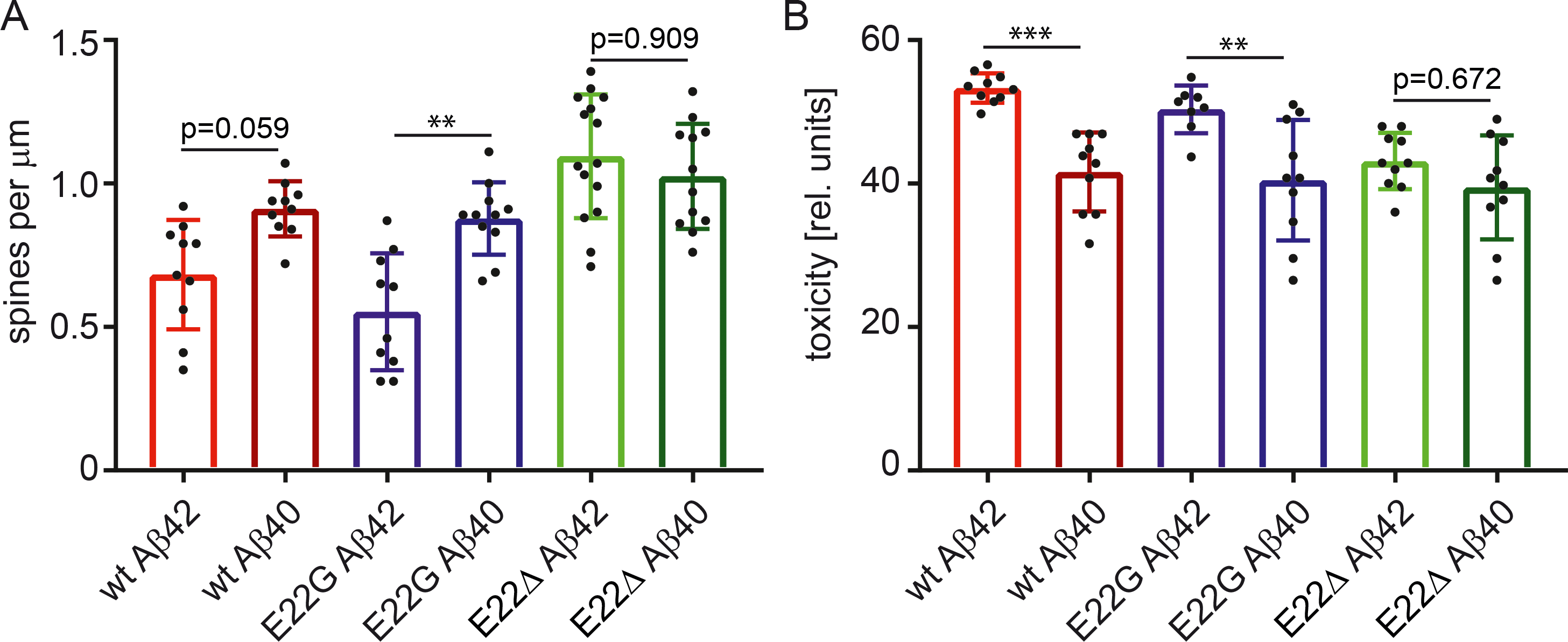

Supplement: S1 Fig — These graphs summarize the findings of Figs 2B and 3B and were plotted to better compare the effects of wt Aβ42, E22G Aβ42 and E22Δ Aβ42 to the respective 40-residue variant, wt Aβ40, E22G Aβ40 and E22Δ Aβ40. A: Spine counts per μm dendrite. Spine density reduction after treatment with wt Aβ42 and E22G Aβ42 was higher compared to wt Aβ40 and E22G Aβ40, respectively. No difference was observed between E22Δ Aβ42 and E22Δ Aβ40. n = 10–15 B: Cytotoxicity in human tau-expressing slices from wild-type mice treated with 1 μM recombinant Aβ. Wt Aβ42 and E22Δ Aβ42 induced more cytotoxicity than wt Aβ40 and E22G Aβ40. No difference was observed between E22Δ Aβ42 and E22Δ s1Aβ40. n = 8–10. Data are means ± SD. Statistical significance was assessed by one-way ANOVA with Tukey's multiple comparison test (**p<0.01, ***p<0.001). (TIF) [file pone.0239584.s002.tif]

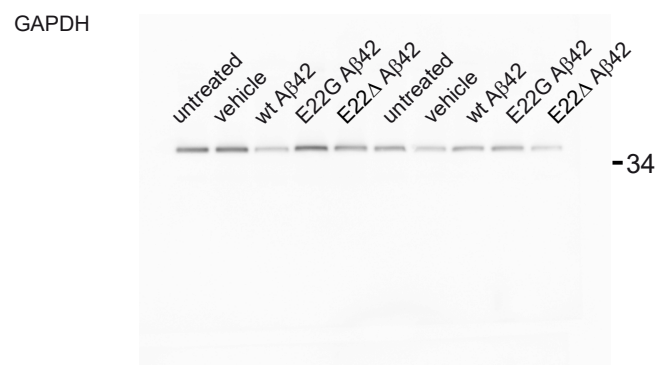

Supplement: S1 File — (PDF) [file pone.0239584.s003.pdf]
